# The “Sweet Spot” of Targeting Tumor Metabolism in Ovarian Cancers

**DOI:** 10.3390/cancers14194696

**Published:** 2022-09-27

**Authors:** Katelyn Tondo-Steele, Karen McLean

**Affiliations:** Division of Gynecologic Oncology, Department of Obstetrics and Gynecology, University of Michigan Medical School, 1500 E. Medical Center Dr., Ann Arbor, MI 48109, USA

**Keywords:** glucose, metabolism, ovarian cancer, glycolysis, fatty acid oxidation, PI3K/AKT/mTOR, oxidative phosphorylation, metabolomics, leptin, insulin

## Abstract

**Simple Summary:**

Ovarian cancer is the most lethal gynecologic malignancy. While most patients will initially respond to treatment, the majority will recur and develop chemoresistance. Therefore, we require a better understanding of how cancer cells evade chemotherapy, including the reprogramming of their signaling pathways in nutrient deficient environments. The aims of this review are to provide an overview of altered metabolism and signaling pathways in ovarian cancer and to outline potential therapeutic modalities to exploit these changes.

**Abstract:**

The objective of this review is to explore the metabolomic environment of epithelial ovarian cancer that contributes to chemoresistance and to use this knowledge to identify possible targets for therapeutic intervention. The Warburg effect describes increased glucose uptake and lactate production in cancer cells. In ovarian cancer, we require a better understanding of how cancer cells reprogram their glycogen metabolism to overcome their nutrient deficient environment and become chemoresistant. Glucose metabolism in ovarian cancer cells has been proposed to be influenced by altered fatty acid metabolism, oxidative phosphorylation, and acidification of the tumor microenvironment. We investigate several markers of altered metabolism in ovarian cancer including hypoxia-induced factor 1, VEGF, leptin, insulin-like growth factors, and glucose transporters. We also discuss the signaling pathways involved with these biomarkers including PI3K/AKT/mTOR, JAK/STAT and OXPHOS. This review outlines potential metabolic targets to overcome chemoresistance in ovarian cancer. Continued research of the metabolic changes in ovarian cancer is needed to identify and target these alterations to improve treatment approaches.

## 1. Introduction

Ovarian cancer is the eighth most common cancer in women worldwide [1]. The majority of ovarian malignancies originate in epithelial cells and are termed epithelial ovarian cancer. High-grade serous carcinoma (HGSC) is the most common histologic subtype and can arise from the Fallopian tube, ovary, or peritoneum. Given the similarities in molecular changes and clinical management for HGSC from these three sites of origin, they will be referred to as “ovarian” cancer throughout this review article. The standard of care for advanced-stage epithelial ovarian cancer is multimodal therapy with debulking surgery and chemotherapy with carboplatin and paclitaxel. Ultimately, most patients with ovarian cancer recur, and their tumors eventually become resistant to platinum-based chemotherapy. There are multiple theorized potential mechanisms for the development of platinum resistance including changes in cell signaling pathways and receptors, gene mutations, alterations in DNA damage repair, and metabolomic alterations [2].

In ovarian cancer, we require a better understanding of how cancer cells reprogram their metabolism both to overcome their nutrient-deficient environment and to become chemoresistant. Noncancer cells rely on mitochondrial oxidative phosphorylation (OXPHOS) to generate adenosine triphosphate (ATP) for energy. During OXPHOS, in the presence of oxygen, glucose undergoes glycolysis to produce pyruvate. Pyruvate is then oxidized to acetyl-CoA, which is the first substrate of the Krebs cycle. The Krebs cycle then leads to oxidative phosphorylation, which yields ATP production. In times of low glucose availability such as fasting, noncancer cells can temporarily switch to fatty acid oxidation for energy production.

In contrast to normal cells, cancer cells develop alternative approaches for ATP production based on the unique conditions of their microenvironment. Despite oxygen availability, cancer cells shift from oxidative phosphorylation and instead rely on increased glycolysis. This is known as the Warburg effect [3]. Cancer cells preferentially utilize glycolytic breakdown of glucose for energy rather than mitochondrial OXPHOS [4]. Glycolysis converts glucose into pyruvate to generate two ATPs as opposed to oxidative phosphorylation, which produces 36 ATPs [5]. As you can see, this is less efficient for cancer cells. This upregulation of glycolysis has been theorized to be an adaptation to the hypoxic tumor environment [6].

Although the Warburg effect notes that cancer cells shift from OXPHOS to glycolysis, increasing evidence has shown an oxidative phosphorylation pathway is still active in cancer cells, including ovarian cancer cells [7]. Even in the presence of oxygen, cancer cells will increase their glucose consumption to produce lactate [7]. More recent data have shown that both of these theories are possible concurrently. Cisplatin resistant ovarian cancer cell lines C200 and PEO4 have been shown to demonstrate metabolic flexibility between glycolysis and OXPHOS [8]. There are several other factors that play a role in the regulation of ovarian cancer metabolism. In addition to glucose, cancer cell metabolism is influenced by fatty acid synthesis [9]. Fatty acids are required for cell membrane proliferation and are an important energy source for cancer cells via β-oxidation [10]. Normally, the only cells able to synthesize fatty acids are hepatocytes and adipocytes. Cancer cells are able to activate this adaptation [10]. De novo synthesis of fatty acids occurs by converting carbon atoms from glucose or amino acids into fatty acids [10]. Under conditions such as hypoxia, cancer cells can upregulate enzymes in order to generate substrates for fatty acid synthesis [10].

There are several pathways that regulate these processes including HIF-1, leptin, glucose transporters, phosphatidylinositol 3-kinase (PI3K)/AKT (also known as protein kinase B), and Janus kinase/signal transducer and activator of transcription (JAK/STAT). The objective of this review is to explore the metabolomic environment of epithelial ovarian cancer that contributes to chemoresistance and to use this knowledge to identify possible targets for therapeutic intervention.

## 2. Metabolomic Alterations in Ovarian Cancer

### 2.1. Glycolysis

Metabolic reprogramming has been considered a hallmark in cancer adaptation and survival [11]. High-grade serous ovarian cancer exhibits high glycolytic activity in regular cell culture conditions [12]. This reliance on glycolysis has been proposed as a therapeutic target for ovarian cancer growth inhibition. Several glycolytic enzymes and transporters in this pathway have been studied as targets, including the glucose transporters GLUT1 and SGLT as well as the glycolytic pathway enzyme hexokinase 2 (HK2).

In order to facilitate increased aerobic glycolysis, cancer cells overexpress glucose transporters including Na+-independent sugar transporters (GLUT) and Na+-dependent sugar cotransporters (SGLT) [13]. GLUT1 is a facilitative glucose transporter that moves glucose into the cell as the first step in the glycolytic pathway [14]. GLUT1 was found to have higher expression in several cancers including HGSC and a significantly higher expression level in advanced stage disease as compared to early-stage disease [13,15,16]. Patients who overexpressed GLUT1 had a shorter disease-free survival. GLUT1 has been demonstrated to be a critical regulator of basal and stress-induced glycolysis in ovarian cancer. When hypoxic stress was introduced to the tumor microenvironment, both GLUT1 protein expression and glycolytic rates were induced [12]. In epithelial ovarian cancer, GLUT1 expression is also positively correlated with tumor proliferation and microvessel density [17].

SGLT is a protein that transports glucose using sodium/potassium ATPase. SGLT can transport glucose across membranes regardless of the glucose concentration in the microenvironment. There are little data on SGLT in ovarian cancer, but SGLT1 has been shown to be an independent biomarker of poor prognosis in ovarian cancer [18]. Additionally, GLUT1 and SGLT1 have been associated with angiogenesis and epidermal growth factor signaling; however, more work is needed to further investigate the relationship between these signaling pathways [12,18].

**Figure 1:**
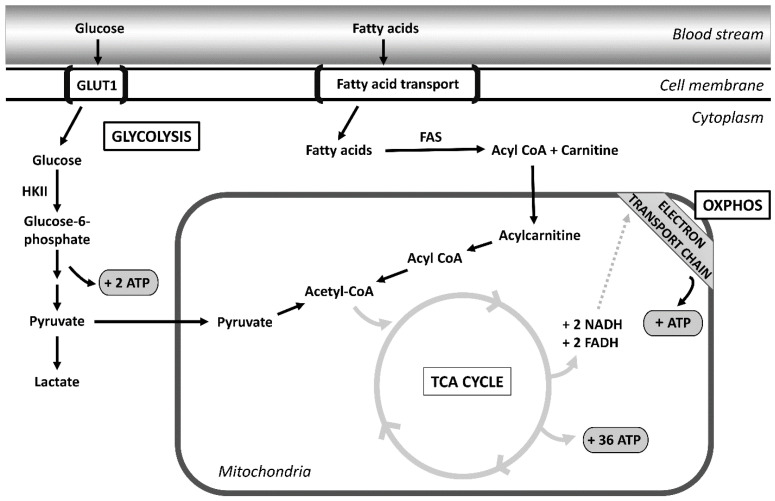
Metabolomic pathways of the cell. Glucose enters the cell through the bloodstream via GLUT1 transporters. Glucose then is broken down by glycolysis producing 2 ATPs and pyruvate. Pyruvate is then transferred to the TCA cycle, which generates NADH and FADH. NADH and FADH transfer their electrons through oxidative phosphorylation (OXPHOS) to generate ATP. Fatty acids are broken down as an alternative form of energy for the cell. Fatty acids enter the cell through membrane transporters. Fatty acids then undergo beta-oxidation, which results in the production of acetyl-CoA. Acetyl-CoA then enters the TCA cycle, which again produces NADH and FADH to use in the OXPHOS pathway. Abbreviations: FAS: fatty acid synthetase; HKII: hexokinase II; ATP: adenosine triphosphate.

HK2 has been described as a key player in cancer [19]. The HK2 enzyme phosphorylates glucose to glucose-6-phosphate and is the rate controlling step of the glycolytic pathway. Glucose-6-phosphate then serves as the precursor to the major next pathways of metabolism including glycolysis, the pentose-phosphate pathway, and the Krebs cycle [19] (Figure 1). HK2 is increased in HGSC relative to non-HGSC [15], and overexpression has been correlated with both chemoresistance and disease recurrence [20]. When ovarian cancer cells are exposed to chronic hypoxia, HK2 expression increases to levels similar to that of multidrug-resistant cells [20]. This has also been demonstrated in other types of cancer cells including glioblastoma and hepatocellular carcinoma, suggesting a role in chemoresistance [20].

### 2.2. Altered Fatty Acid Metabolism

It is commonly known that glucose plays a role in cancer metabolism; however, it is less well known that cancer cells are influenced by other metabolic pathways such as increased fatty acid synthesis. Noncancer cells use dietary lipids from the bloodstream, while cancer cells show increased rates of de novo fatty acid biosynthesis [21,22]. One possible reason for this increase in biosynthesis is as a protective mechanism for cancer cells in the nutrient-deprived and oxygen-depleted tumor microenvironment [21]. When the supply of fatty acids is scarce, cancer cells are supported by fatty acid synthesis [21,22]. It is thought that the availability of lipids in the microenvironment can fuel tumor growth [23].

Many of the enzymes important for fatty acid synthesis are abnormally expressed in ovarian cancer cells. One important enzyme is fatty acid synthase (FASN), which works by converting excess carbohydrates into fatty acids. These fatty acids are broken down through β-oxidation to use for energy. FASN expression in normal adult tissues is generally very low, but in many types of cancer it is significantly upregulated, which correlates with poorer prognosis [24]. FASN is regulated by hormones, growth factors, and diet; it is upregulated in rapidly proliferating cells [25]. FASN has been shown to be highly expressed in ovarian cancer tissue, with higher expression linked to decreased survival rates [25]. As more rapidly proliferating cells develop increased energy demands, FASN is upregulated. Thus, some studies have suggested using FASN as a metabolic marker for ovarian cancer cell proliferation [24].

Cisplatin-resistant ovarian cancer cells have been shown in vitro to undergo reprogramming from glucose dependence to increased fatty acid uptake [26]. One explanation for this increase in fatty acid synthesis is the cellular need to enhance energy production through β-oxidation. Fatty acid oxidation allows for production of reduced nicotinamide adenine dinucleotide phosphate (NADPH) and ATP [27]. The production of NADPH helps counteract oxidative stress, as inhibition of fatty acid oxidation results in the accumulation of reactive oxygen species and eventual cell death [27]. The metabolic benefits of fatty acid oxidation have been hypothesized as one reason for the predilection of HGSC to metastasize to fat-rich tissues within the abdomen such as the omentum so that they can maintain energy demands through the tumor microenvironment resources [28].

### 2.3. Oxidative Phosphorylation

Most ovarian cancer cells maintain both glycolytic and OXPHOS pathways [8]. OXPHOS involves a series of oxidation-reduction reactions that result in the phosphorylation of adenosine diphosphate (ADP) to produce ATP. OXPHOS allows for ATP production at very low oxygen levels. High OXPHOS in ovarian cancer cells has been demonstrated to use mainly fatty acids and glutamine, similar to metabolic changes also seen in other solid tumors [29].

It has been shown that OXPHOS status is associated with response to chemotherapy. Ovarian cancers that demonstrate defects in the homologous recombination pathway generally demonstrate platinum sensitivity [30] and are noted to have higher OXPHOS activity following tumor inhibition by platinum-based chemotherapy in a mouse model [29]. In contrast, in homologous recombination proficient ovarian cancers, there was stronger growth inhibition in patient-derived xenograft models with higher as compared to lower OXPHOS levels [29]. Additionally, in patient tumor samples, it was noted that patient survival was improved in those who had high OXPHOS status [29].

Finally, chemoresistance has been linked to an increased dependence on OXPHOS [8]. Cisplatin-resistant ovarian cancer cell lines OVCAR5, OVCAR3 and OVSAHO display the ability to utilize both glycolysis and OXPHOS [31]. These cells were noted to be metabolically diverse and able to evade cell death by maintaining energy production through multiple pathways [8]. Platinum treatment was demonstrated to increase mitochondrial OXPHOS activity and OXPHOS gene expression, suggesting these cells utilize OXPHOS for survival [31].

### 2.4. Hypoxia-Induced Factor

Hypoxia is considered a hallmark of cancer and plays a major role in the metabolic reprogramming of tumor cells [11]. Hypoxia is a stress signal to cells, leading them to respond by increasing glycogen content [32]. This process is regulated primarily by hypoxia-induced factor (HIF) signaling [32,33]. The ability to increase glycolysis during conditions of hypoxia allows decreased mitochondrial use, which in turn lessens free radical damage and apoptosis caused by mitochondria-mediated reactive oxygen species [34]. This allows for a continued energy supply for cancer growth in the hypoxic tumor microenvironment.

The majority of studies in ovarian cancer have indicated that elevated HIF-1α levels are a predictor of a poor prognosis [35]. HIF-1α has been associated with tumor angiogenesis and ovarian cancer aggressiveness [36]. HIF-1α loss of function results in decreased tumor growth, vascularization, and metastasis, whereas HIF-1α gain of function has the opposite effects [33].

There are several mechanisms that allow HIF to affect cancer metabolism. As described above, glycolytic enzymes are the gatekeepers of the glycolytic pathway. HIF-1α stimulates glycolysis through induction of GLUT1, HK2, and other glycolytic enzymes [17,33]. This increase in glycolytic enzymes facilitates increased glucose uptake into cells and provides metabolic requirements to tumor cells [17]. HIF-1α also induces invasion and metastasis of ovarian cancer cells through the activation of the rate-limiting enzymes of the glycolysis pathway, hexokinase 2 and phosphofructokinase, to promote the Warburg effect [35].

A second function of HIF-1α is the ability to inhibit p53 transcriptional activity, and therefore to block p53-mediated apoptosis [35,37]. This may also contribute to chemoresistance, as cisplatin targets the p53-dependent apoptotic pathway in ovarian cancer cells [38]. High HIF-1α expression has been demonstrated to downregulate other targets such as estrogen receptor alpha and thus reduce the effect of antiestrogen therapies including tamoxifen [39]. HIF-1α can induce carbonic anhydrase, which converts carbon dioxide and water into bicarbonate and protons. The proton byproduct of this reaction causes a change in the pH of the environment; this shift decreases the absorption and concentration of drugs within the cell, thereby decreasing the potential actions of that drug [4].

HIF-1α regulates the expression of additional genes that promote tumor progression including vascular endothelial growth factor (VEGF), which is a pro-angiogenic factor that promotes neovascularization [40]. Elevated VEGF levels are associated with increased angiogenesis and poorer survival in epithelial ovarian cancer [41]. VEGF is a downstream gene of HIF-1α and expression is increased when HIF-1α is induced. Hypoxia induces a significant increase in both HIF-1α and VEGF in ovarian cancer [40]. Silencing HIF-1α resulted in reduction in angiogenesis even in hypoxic conditions, making this a potential therapeutic target [40].

### 2.5. Leptin

Leptin is a peptide hormone that regulates appetite, body composition, metabolism, and reproductive function [42]. It is synthesized mainly in white adipocytes [2]. Adipose tissue can be divided into white and brown fat [43]. White adipose tissue is used for energy storage, body insulation, and secretion of adipokines [43], while brown adipose tissue contains high amounts of mitochondria and mediates non-shivering thermogenesis. Brown adipocytes generate heat and maintenance of body temperature homeostasis [43].

Elevated leptin levels are associated with poorer prognosis in ovarian cancer [2]. In an analysis of patient tumor samples, overall survival was significantly decreased in the leptin-high expression group compared with the low expression group [2]. This was echoed in The Cancer Genome Atlas (TCGA) in which patient with tumors expressing higher leptin levels had worse progression-free and overall survival rates [44]. Leptin has also been associated with chemoresistance in epithelial ovarian cancer [2]. Leptin was noted to reduce the proportion of cells in the G2/M phase in HO8910PM and OV-MZ-15 ovarian cancer lines cells treated with paclitaxel, suggesting that leptin may reduce the sensitivity of ovarian cancer cells to paclitaxel’s effect on microtubules [2]. Leptin was also able to confer chemoresistance through leptin-induced expression of epithelial-mesenchymal transition (EMT) genes [2,44]. Epithelial cancer cells must undergo EMT in order to migrate and metastasize [44]. In patients who had undergone paclitaxel chemotherapy, high leptin expression was associated with activation of EMT gene enrichment, suggesting high leptin expression may lead to paclitaxel resistance through activation of EMT in ovarian cancer [2].

Insulin is a primary regulator of leptin production via glucose metabolism [42]. Leptin binds to obesity (Ob) receptors, leading to activation of JAK/STAT, PI3K, and MAP kinase signaling [45,46]. Leptin can then induce tumor progression and metastasis through these signaling pathways in obesity-related tumors [46]. Through the activation of JAK/STAT and PI3K pathways, leptin stimulates ovarian cancer cell growth and prevents cell apoptosis [47]. Leptin was demonstrated to increase ovarian cancer growth in a dose-dependent manner in vitro [47]. Furthermore, leptin showed an anti-apoptotic effect on ovarian cancer cells in vitro by inhibiting poly (ADP-ribose) polymerase (PARP) cleavage [47]. Additionally, leptin has been shown to stimulate matrix metalloproteinase, cyclin D1, and Mcl-1 protein expression to induce cell invasion and tumor progression [46,47,48]. The increase in Mcl-1 protein levels is associated with poorer prognosis in ovarian cancer [47].

Leptin also plays a role in the immune response, angiogenesis, and lipolysis [42,45] and increases systemic inflammation (TNF-α, IL6), promoting angiogenic factors (VEGF) and increased hypoxia-inducible factor-1a (HIF-1) expression [45]. These changes promote cancer cell survival, proliferation, and migration as discussed above [45].

### 2.6. Insulin-like Growth Factor Binding Proteins

Insulin-like growth factors (IGF) are polypeptide hormones that are produced in the liver in response to growth hormone [49]. IGFs play a role in endocrine and paracrine functions to promote cell growth, proliferation, survival, and apoptosis [49,50]. IGF binds to insulin-like growth factor receptor 1 (IGF-1R), which activates signaling for cell growth and reproduction. IGF-1R has been associated with various outcomes in several cancers including breast cancer [51]. In ovarian cancer, higher IGF-1 expression was associated with disease progression [51].

IGF signaling is regulated by the six IGF-binding proteins (IGFBP) [50]. IGFBPs affect growth and metabolism and have been linked to Phosphatase and tensin homolog (PTEN)/PI3K signaling [52]. IGFBP-2 and IGFBP-3 are the most common binding proteins involved in ovarian cancer metabolism. IGFBP-2 has been shown to correlate with the aggressiveness of several cancer types, including overexpression in HGSC [53]. High serum levels of IGFBP-2 correlated with higher stage ovarian cancer and poorer prognosis [53,54]. In preclinical studies, when IGFBP-2 was added to ovarian cancer cells, increased cell proliferation was noted and multiple MAP kinase signaling pathways were activated including Raf, MEK, ERK1/2, and Elk-1 [55]. Blocking these MAP kinase pathways with single agent inhibitors then prevented phosphorylation of ERK1/2 and cell proliferation, suggesting that IGFBP signaling promotes ovarian cancer growth through several cross-talking pathways [55].

Increased IGFBP-3 expression has been demonstrated to negatively correlate with the invasiveness of ovarian cancer in vitro and in vivo [56]. Expression of IGFBP-3 inhibited tumor migration, while suppression of IGFBP-3 with siRNA resulted in resumption of migration activity, suggesting IGFBP-3 has a role in the regulation of cell migration [56]. There is also evidence showing that IGFBP-3 mediates HIF expression during prolonged hypoxia [56]. Hypoxia reduces the expression of IGFBP-3, which then activates HIF-2α expression. IGFBP-3 was demonstrated to inhibit angiogenesis through this pathway and could be a potential diagnostic marker or target for therapy [56]. Collectively, these effects accelerate ovarian cancer growth.

## 3. Signaling Pathways

### 3.1. PI3K/AKT/mTOR

**Figure 2:**
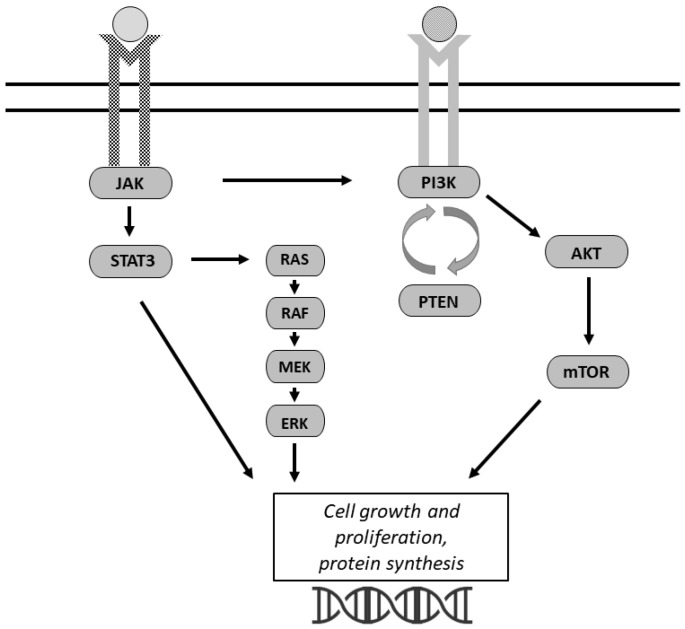
Signaling pathways altered in ovarian cancer cells. There is extensive crosstalk between the pathways depicted. The JAK/STAT signaling pathway and the PI3K/AKT/mTOR signaling pathway both regulate cell growth and proliferation. The JAK/STAT pathway activates PI3K as well as the MAPK signaling to stimulate downstream growth. The pathways shown also impact angiogenesis and cell migration.

The PI3K/AKT/mammalian target of rapamycin (mTOR) pathway is one of the most commonly activated pathways in human cancers (Figure 2) [57]. PI3K is a cytoplasmic signaling enzyme that propagates receptor-mediated signals from growth factors and cytokines to activate downstream effectors including AKT and mTOR [58]. The PI3K/AKT/mTOR pathway regulates glycolysis, lipid synthesis, the tricarboxylic acid (TCA) cycle, and OXPHOS [59]. This pathway has been well-studied pertaining to cancer growth and glucose metabolism [34,57]. PI3K inhibits pyruvate kinase 2, a rate-limiting enzyme of glycolysis, as well as the pentose phosphate pathway through stabilization of glucose-6-phosphate dehydrogenase [59]. These steps directly play a role in the Warburg effect by altering glucose delivery to the cells [59]. AKT activation promotes glucose uptake, glycolytic flux, and lactate excretion [57].

Several of the proteins already discussed in this review utilize PI3K for signaling including GLUT1, HK2, FASN, and HIF-1α. GLUT1, as noted above, is highly expressed in cancer cells; this elevated expression level is associated with abnormal activation of the PI3K/AKT pathway in cancer cells [60]. AKT and mTOR signaling increase glucose uptake through GLUT1 on the plasma membrane, which then drives glucose into the cell and enhances the Warburg effect [61,62]. The PI3K pathway promotes glycolysis through AKT signaling and activating downstream mTOR, which then phosphorylates HK2, controlled at the transcriptional level by HIF-1α [62]. The PI3K/AKT/mTOR pathway is also a regulator of fatty acid synthesis through activation of ATP citrate lyase (ACLY) and FASN, which allows for de novo lipid synthesis [62]. During nutrient shortages, FASN undergoes metabolic reprogramming via alterations in PI3K/AKT/mTOR signaling [63].

Mutations in the PI3K/AKT/mTOR pathway have been identified in ovarian cancers through TCGA [64]. Deregulation of this pathway has been demonstrated to facilitate chemoresistance in ovarian cancer through several mechanisms. G6PD, which is stabilized through PI3K, has been shown to have higher expression in the cisplatin-resistant ovarian cancer cell line C13* than the cisplatin-sensitive line OV2008 [65]. miRNAs have also been associated with ovarian cancer chemoresistance. For example, miR-93 has been demonstrated to promote cell growth and chemoresistance to cisplatin by targeting PTEN and phosphorylation of AKT [63].

Salt-induced kinase 2 (SIK2) is an AMP-activated protein kinase (AMPK)-related protein kinase that plays a role in cellular metabolism through the PI3K pathway. A hypoxic environment induces expression of HIF-1α through phosphorylation of AKT/mTOR, promoting cell proliferation [35]. In ovarian cancer, SIK2 upregulates HIF-1α, which then increases glycolytic enzyme HK2 translation [9,35]. Furthermore, silencing of HIF-1α downregulated AKT/mTOR in ovarian cancer cells, further demonstrating the interplay between these two pathways. SIK2 also has the ability to enhance fatty acid synthesis and promote proliferation of cells in ovarian cancer cells through the PI3K pathway [9].

### 3.2. JAK/STAT

Multiple cytokine and growth factor signals exert their intracellular effects though the activation of receptor-associated JAK [66,67]. Activated JAK then phosphorylates STAT factors to trigger intracellular signaling cascades [67,68]. The JAK/STAT pathway plays a critical role in normal cellular homeostasis and metabolism.

JAK/STAT has been detected in a wide variety of human cancer cells and has been shown to play a role in several oncogenic processes [67]. STAT3 has been demonstrated to act as a mediator of aerobic glycolysis. Tyrosine phosphorylation of STAT3 through JAK promotes oncogenic transformations including aerobic glycolysis and reduces mitochondrial respiration [69]. STAT3 also induces HIF-1α, which upregulates glycolytic processes [69]. The JAK/STAT pathway also plays a key role with insulin in regulating IGF-1 [66].

JAK/STAT alters leptin receptor binding to regulate food intake and energy expenditure [66]. As previously described, leptin induces cell proliferation and inhibits apoptosis in ovarian cancer. Leptin enhances cell growth through PI3K, as well as JAK2. Phosphorylation of JAK2 is the first signal after leptin binds to its receptor [47]. JAK2 triggers downstream pathways PI3K and MEK/ERK1/2 [47], which then stimulate cell growth and inhibition of apoptosis [47].

Finally, the cytokine interleukin-6 (IL-6) signals through the JAK/STAT pathway, and IL-6 overexpression in obese patients has been shown to mediate insulin resistance [66]. IL-6 is involved in many tumor types and has been found to be highly expressed in patients with ovarian cancer [70]. Overexpression of IL-6 can promote proliferation and invasion of ovarian cancer cells and has been associated with poorer prognosis [70]. IL-6 can also induce HIF-1α activity via JAK/STAT signaling, which has been shown to be associated with chemoresistance to cisplatin in ovarian cancer [71].

The JAK/STAT pathway has been shown to be activated in epithelial ovarian cancer and activation of STAT3 is associated with platinum resistance [72]. It has also been shown as a prognostic factor in ovarian cancers, especially clear cell subtypes [73].

## 4. Targeted Therapies in Ovarian Cancer

Given the importance of the metabolomic environment and cancer reprogramming of glucose metabolism, multiple preclinical studies and clinical trials have investigated different treatment strategies that target the metabolic pathways discussed herein. The compounds listed in Table 1 are from preclinical or ongoing clinical trials, with a focus on the targets, rather than a comprehensive list of all compounds.

Inhibitors of glycolysis include GLUT1 inhibitors, G6PD inhibitors, and metformin [12,65]. Inhibitors of fatty acid metabolism include FASN inhibitors. HIF-1α is targeted by 2-Methoxyestradiol (2ME2) and EP0057. 2ME2 is an endogenous metabolite of estradiol that destabilizes microtubules and exerts anti-angiogenic properties through the inhibition of HIF-1α [74]. In the phase II trial, the clinical benefit rate for patients with platinum-resistant epithelial ovarian cancer treated with single agent 2ME2 was 31.3% [74]. EP0057 is a nanoparticle-drug conjugate of camptothecin and the sugar molecule cyclodrexin that has been shown to inhibit HIF-1α. This was a Phase Ib/II trial of EP0057 with weekly paclitaxel in heavily pre-treated patients with recurrent ovarian cancer. There was a 31.6% overall response rate, including one complete response, and a 5.4-month median progression-free survival (NCT02389985). EP0057 is now being tested in combination with olaparib in a clinical trial (NCT04669002). There are several ongoing clinical trials for PI3K/AKT/mTOR inhibitors. Clinical trial NCT01623349 combines PI3K inhibitors with olaparib in high-grade serous ovarian cancer, while trial NCT01068483 is looking at PI3K inhibitors as monotherapy. Although there are no current clinical trials targeting IGF-1R, prior phase II trials have shown IGF-1R inhibition did not improve progression-free survival in the upfront setting (NCT00718523) and demonstrated modest activity in the recurrent setting (NCT00719212). There are several preclinical studies looking at the JAK/STAT pathway as a potential target in ovarian cancer. A JAK2 inhibitor, CYT387, in combination with paclitaxel, resulted in the suppression of JAK2/STAT3 activation which then coincided with significantly smaller tumors in mice [68]. Combining targeted agents may be a strategy to overcome resistance mechanisms, as clearly these signaling pathways are highly interconnected.

**Table 1:** Inhibitors targeting the ovarian cancer metabolism network in preclinical models and clinical studies.

Targeted Pathway	Specific Agent or Compound Name	Trial Phase	Findings/Summary	Study or Trial Number
Glycolysis	miR-206, miR-613	Preclinical	G6PD inhibitors, miR-206, and miR-613 sensitize resistant cells to cisplatin.	Zheng et al. [65]
	GLUT1 inhibitor BAY-876	Preclinical	Targeting of GLUT1 suppresses glycolytic metabolism and in vitro and in vivo ovarian cancer growth.	Ma et al. [12]
	Metformin	Clinical trial	Role of combining carboplatin, paclitaxel, and metformin in advanced stage ovarian cancer. Study is ongoing.	NCT02437812; Brown et al. [75]
Fatty acid metabolism	FASN inhibitor Compound 34	Preclinical	Compound 34 inhibits cell proliferation in multiple cancer cell lines including ovarian, prostate, lymphoma, lung, and breast.	Lu et al. [76]
	FASN inhibitor cerulenin	Preclinical	FASN inhibitor cerulenin strongly blocked FASN protein expression and both stimulated apoptosis and re-induced platinum sensitivity.	Bauerschlag et al. [77]
Oxidative phosphorylation	Respiratory chain complex I inhibitor IACS-010759	Preclinical	Inhibitor caused mitochondrial swelling and ATP depletion to delay cancer progression and prolonged the lifespan of ovarian cancer PDX tumors.	Ghilardi et al. [78]
Hypoxia	2-Methoxyestradiol (2ME2)	Phase II clinical trial, completed	2ME2 is an endogenous metabolite of estradiol that destabilizes microtubules and exerts anti-angiogenic properties; 31.3% clinical benefit rate in ovarian cancer.	Matei et al. [74]
	Camptothecin nanoparticle-drug conjugate (NLG207/CRLX101/EP0057)	Phase Ib/II clinical trial, completed	HIF-1α inhibition in combination with weekly paclitaxel yielded 31.6% overall response rate.	NCT02389985
	EP0057, a nanoparticle-drug conjugate (NDC) of camptothecin	Clinical trial	EP0057 in combination with olaparib in ovarian cancer. Study is ongoing.	NCT04669002
PI3K/AKT/ mTOR	Oral AKT inhibitor GSK2141795	Clinical trial	Pharmacokinetics and pharmacodynamics study in ovarian cancer patients. Study is ongoing.	NCT01266954
	Oral PI3K inhibitors BKM120 or BYL719	Phase I clinical trial	PI3 kinase inhibition in combination with olaparib.	NCT01623349
	Oral PI3K inhibitor BKM120	Phase I clinical trial	Safety of BKM120 monotherapy in advanced solid tumors.	NCT01068483
JAK/STAT	JAK2-specific inhibitor CYT387	Preclinical	CYT387 in combination with paclitaxel resulted in the suppression of JAK2/STAT3 activation, which coincided with significantly smaller tumors in mice.	Abubaker et al. [68]

## 5. Conclusions

In summary, there are multiple critical pathways in the metabolomic environment that are deranged in ovarian cancer. Alterations in glucose metabolism including the Warburg effect have been described in ovarian cancer and have been linked to chemoresistance. Cancer cells are able to reprogram their glycogen metabolism in several ways including alterations in glycolysis, fatty acid metabolism, and oxidative phosphorylation to overcome their nutrient-deficient environment. This allows continued tumor growth and the development of chemoresistance. The metabolic changes observed in ovarian cancer are facilitated by signaling pathways PI3K/AKT/mTOR and JAK/STAT. Within these pathways are factors HIF-1α, IGFBPs, GLUT1, FASN, and leptin, which have all been demonstrated to be potential therapeutic targets in ovarian cancer. Several emerging studies support the role of targeting these pathways. Thus, we continue to require a better understanding of how cancer cells reprogram their metabolism to overcome their nutrient-deficient environment and become chemoresistant in order to target these alterations for improved treatment strategies.
